# Assessing the physiology of weight loss based on real-time weight monitoring: The ADAPT behavioral weight loss study protocol

**DOI:** 10.1371/journal.pone.0354678

**Published:** 2026-08-25

**Authors:** Susan J. Melhorn, Ana P. Valencia, Jeannette M. Schenk, Jose M. Garcia, Sarah J. Beatty, Anne Konchan, Arthur F. Brandao, Natalia L. Acosta-Vega, David J. Marcinek, Jenny Tong, Marian L. Neuhouser, Ellen A. Schur

**Affiliations:** 1 Department of Medicine, University of Washington, Seattle, Washington, United States of America; 2 Fred Hutchinson Cancer Center, Seattle, Washington, United States of America; 3 Geriatric Research Education and Clinical Center, Veterans Affairs Puget Sound Health Care System, Seattle, Washington, United States of America; 4 Department of Radiology, University of Washington, Seattle, Washington, United States of America; 5 Lilly Corporation, Indianapolis, Indiana, United States of America; PLOS: Public Library of Science, UNITED STATES OF AMERICA

## Abstract

Improving health outcomes via sustained weight loss and maintenance requires advancing understanding of the metabolic, appetitive, and neurological alterations that counteract—and eventually halt—weight loss. Prior studies have demonstrated increased appetite as well as metabolic adaptations, such as increased energy efficiency, in response to weight loss. However, previous study designs utilized experimentally determined weight loss plateaus and did not investigate spontaneously occurring plateaus nor study participants in their natural, free-living, environments during weight loss. The *Assessing Diet, Appetite, and Physiology Throughout weight loss* (ADAPT) study was designed to address these limitations of prior research and elucidate mechanistic factors involved in spontaneous cessation of intentional weight loss in humans. ADAPT enrolls participants with obesity who undergo behavioral weight loss via a reduced calorie diet and increased physical activity. Participants’ daily weight is monitored remotely, and new analytic approaches identify weight-loss phases in real time so that study assessments can be targeted to each participants’ individualized trajectory during dynamic weight loss. Deep phenotyping of participants includes anthropometric, metabolic, neurophysiologic, and behavioral assessments; physical activity and physiologic monitoring via wearable devices; and serial sampling of biological tissues such as blood, adipose tissue, and muscle. In summary, the ADAPT study design is generating a uniquely informative and integrative dataset for deepening understanding of the biological mechanisms that halt behaviorally-induced weight loss and thereby limit its long-term health benefits for patients with obesity. NCT06174389.

## Introduction

Behavioral strategies for obesity treatment reliably result in weight loss and health benefits [1–3]. However, weight loss typically plateaus at ~7–10% of total body weight (a plateau phase [4]) and weight regain is common [4–6] limiting the ability of behavioral intervention strategies to modify obesity-related disease risk in a sustained manner. Improving health outcomes via sustained weight loss and maintenance requires advancing understanding of the metabolic, appetitive, and neurological alterations that counteract—and eventually halt—weight loss. Prior research has documented increased hunger, decreased satiety [7,8] and increased food intake after a period of weight loss [9]. Additional findings [10] demonstrate reduced satiety response in brain regions controlling reward and motivation, which may contribute to the biology of weight loss plateaus. Documented metabolic responses to weight loss include declines in total body [11–14] and resting energy expenditure [11,15,16] and increased skeletal muscle work efficiency [17,18].

Additionally, weight loss is a dynamic and individual process, but prior study designs have not adequately captured these characteristics. Early phase weight loss has been previously defined as rapid, of 4–6 weeks’ duration, and reflective of shifts in body water content more so than adipose reduction [19]. Active weight loss involves steady adipose depot depletion that eventually ends at a weight loss plateau (also called static phase [20]). Weight loss plateaus, while individually variable, reflect cessation of weight loss, are frequently involuntary, and are documented consistently in behavioral weight loss trials [4,5], chronic caloric restriction [21], pharmacologic studies [4,22], and bariatric surgery [23]. Physiologic adaptations that can be accentuated at the plateau phase include appetite stimulation [24,25] and reductions in resting energy expenditure (REE) [12,24,26,27]. Prior studies of weight-reduced plateaus have typically halted weight loss at an experimentally determined weight and then maintained a steady weight by prescribing participants a calorically controlled liquid maintenance diet [11,17,18,28–31]. These controlled designs are limited by not accurately characterizing the physiologic and neurologic adaptations opposing further weight loss at a spontaneously occurring plateau and are also unable to elucidate factors predicting cessation of weight loss since it is experimentally determined. Further, in recent years, the capability to remotely collect real-time data during weight loss has advanced, but investigations of behavioral weight loss have yet to fully exploit these technologies. Study designs that adaptively assess physiological changes in multiple tissue types as the pace and extent of weight loss evolve over time could provide novel insights into the driving factors underlying weight loss plateaus in humans.

The *Assessing Diet, Appetite, and Physiology Throughout weight loss* (ADAPT) study was designed to address limitations of prior research. Novel approaches were integrated into the protocol, including multi-modal phenotyping of appetite and energy expenditure through biological sampling and neuroimaging in combination with behavioral assessments. Importantly, by using remotely assessed daily weights to characterize each participant’s weight-loss trajectory, ADAPT individualizes timing of biological assessments according to defined phases of weight loss, including a weight-loss plateau. This adaptive design and phase-based approach distinguishes ADAPT from prior studies that relied on fixed, predetermined time points.

**Fig 1:**
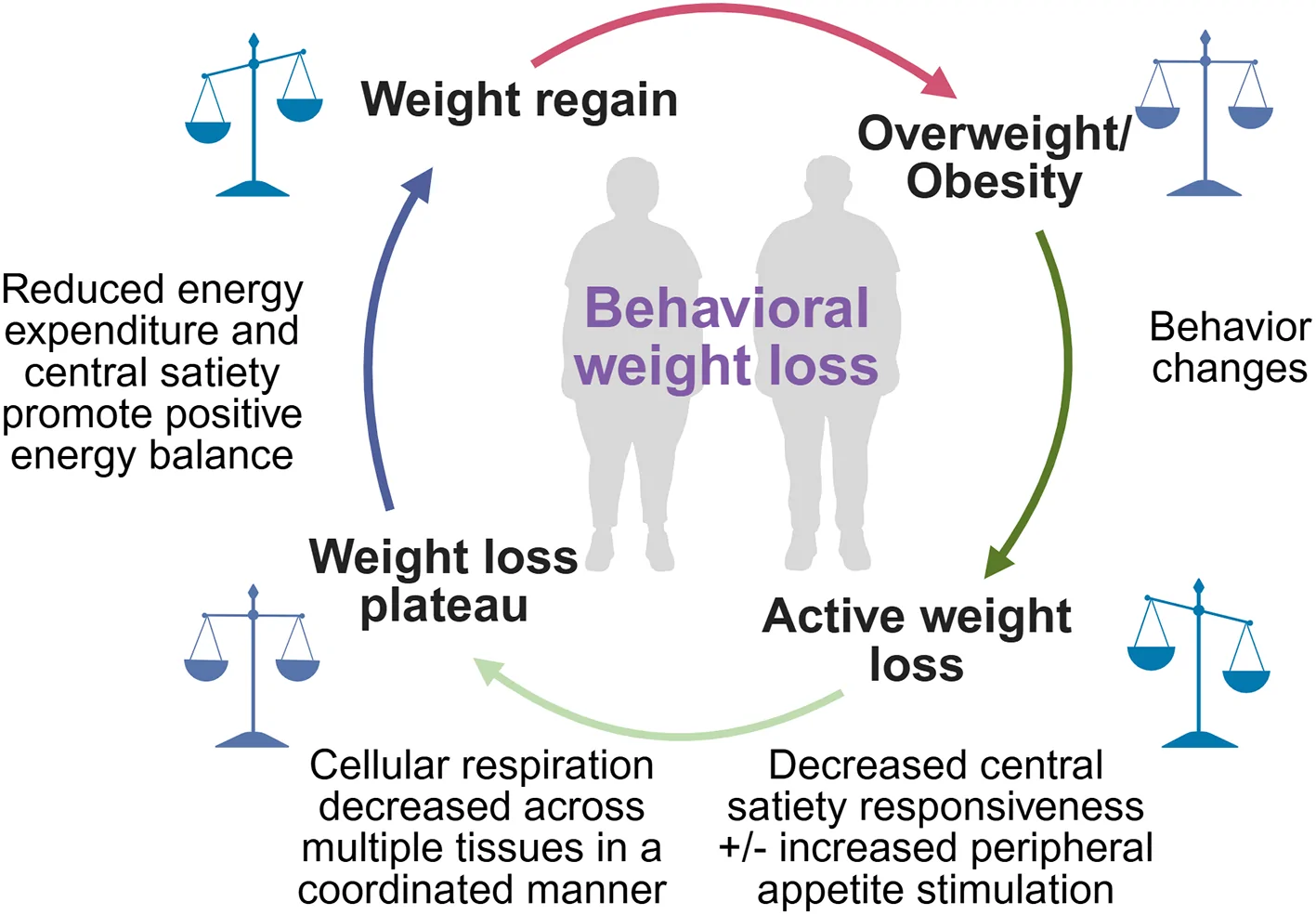
Conceptual model of the ADAPT study. Behavioral weight loss involves lifestyle modifications sufficient to cause negative energy balance and weight loss. A substantial percentage of individuals undergoing behavioral weight loss will experience a weight loss plateau—an involuntary cessation of weight loss and restored energy balance at a reduced weight. The ADAPT study is designed to assess physiological changes during weight loss in real-time. The hypothesized changes in energy efficiency and appetite could make achieving additional weight loss more challenging and promote involuntary weight regain.

The overarching hypothesis of ADAPT is that an involuntary weight loss plateau is characterized by accentuated changes in bioenergetics and neuro-physiology that thwart efforts at additional weight loss and, ultimately, favor weight regain [Fig 1]. ADAPT also investigates predictors of entering into a weight loss plateau that could be mechanistically related to cessation and/or the degree of weight loss in response to behavioral weight loss. To investigate these hypotheses, the study implements a remotely delivered group behavioral weight loss program augmented by individual support to stimulate weight loss. We combine this program with real-time monitoring and individually timed outcome assessments to study 90 adults with obesity at baseline, and re-evaluating those who enter active weight loss and plateau phases, as defined by the study protocol. The specific aims of this research are threefold: 1) to test if an involuntary weight loss plateau is distinguished metabolically from other phases of weight loss by alterations in cellular bioenergetics across multiple tissue types, 2) to test for neurophysiologic changes consistent with reduced satiety at a weight loss plateau and their relation to metabolism, and 3) to explore predictors of a weight loss plateau. We hypothesize that peripheral blood mononuclear cells, myofibers and adipocytes obtained during the plateau phase will show reductions in mitochondrial respiratory capacity and/or improved coupling relative to baseline and active weight loss. We further hypothesize that the central satiety response will be reduced from baseline at a weight loss plateau, activation in inhibitory control regions involved in self-regulation will be enhanced, and both will correlate with suppressed tissue-level and whole-body metabolism [Fig 1]. Finally, we hypothesize that our exploratory predictive analyses will identify potential behavioral, neurophysiologic, and bioenergetic predictors of early entry into an involuntary weight loss plateau that would be appropriate targets for future research or interventions.

## Materials and methods

### Study design and setting

**Fig 2:**
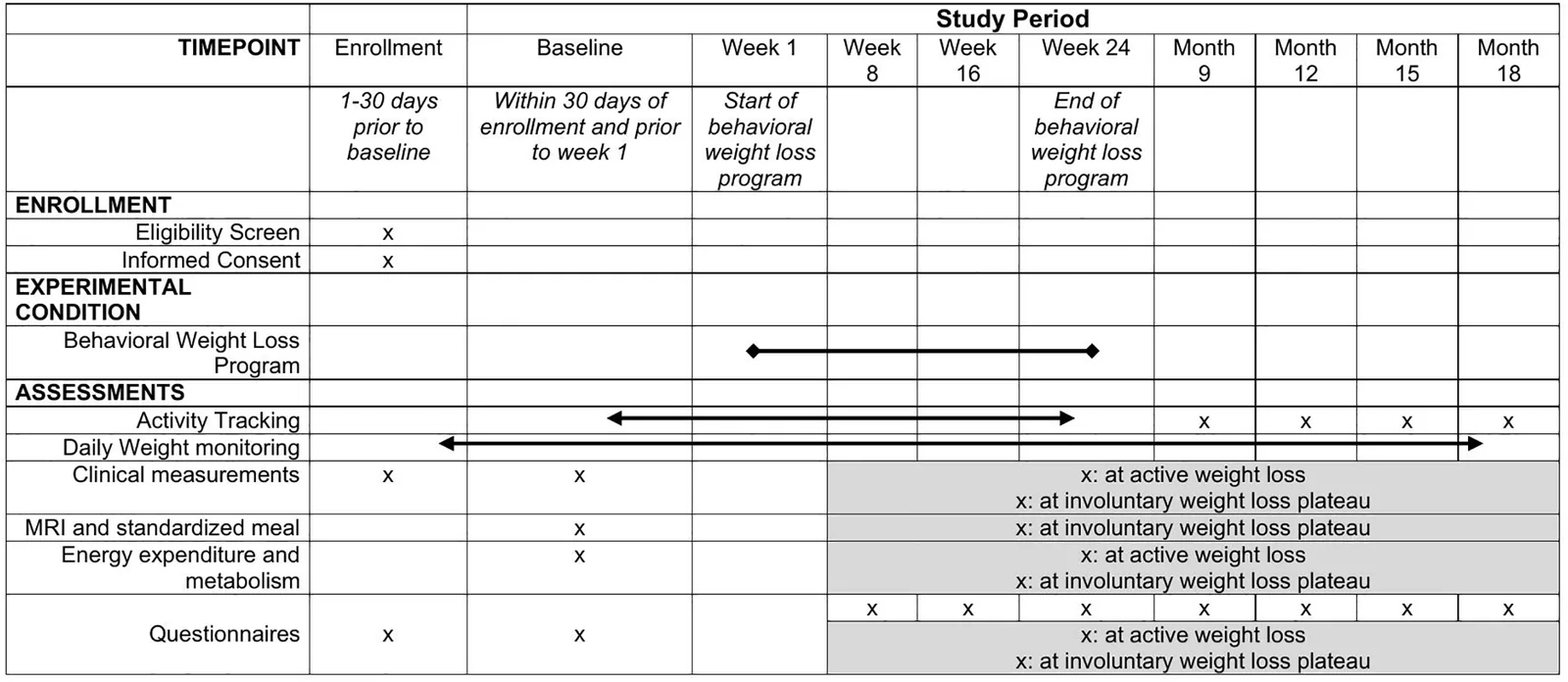
SPIRIT schedule of enrollment and assessments. Assessments in gray boxes occur at individualized time points aligned with phases of weight loss.

**Fig 3:**
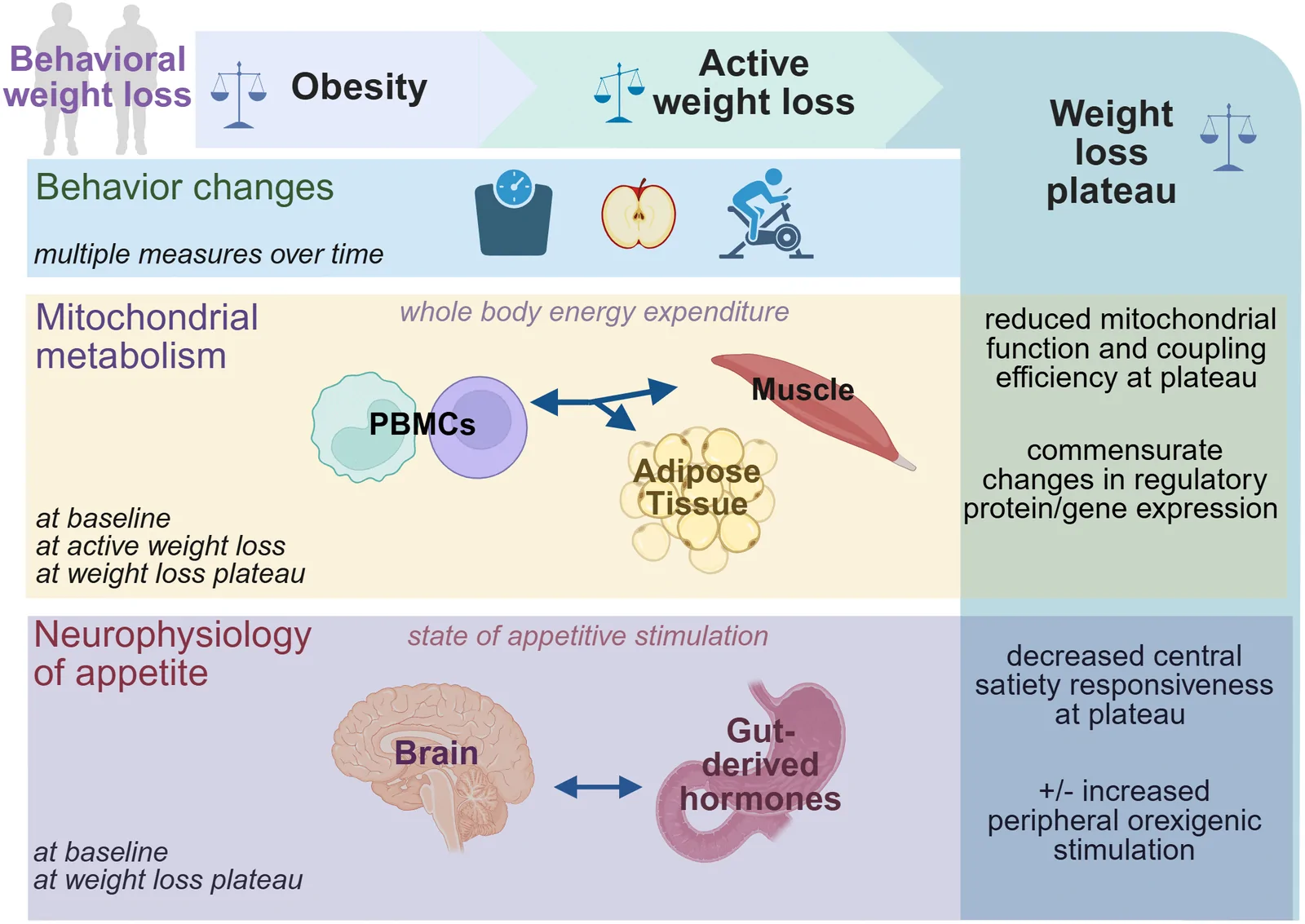
Mechanisms of physiological changes associated with weight loss plateau are investigated in the ADAPT study. A behavioral weight loss program is used to evoke entry into different phases of weight loss at which mechanistic outcomes will be compared. Phenotyping for the state of energy expenditure includes obtaining peripheral blood mononuclear cells (PBMCs), muscle (myofibers), and adipose tissue specimens, as well as measures of whole-body resting energy expenditure by indirect calorimetry. We will examine each tissue type individually and also investigate how tissue “cross talk” among PBMCs (specifically T-cells), muscle, and adipose tissue contributes to systemic changes in bioenergetics. To assess the neurophysiology of appetite, brain responses in regions governing appetite, reward, and self-regulatory capacity are assessed by functional magnetic resonance imaging (fMRI) before and after a meal. fMRI is used to assess changes in the central satiety response at a weight loss plateau as well as activation in inhibitory control regions. Relationships of neurophysiologic outcomes with eating behavior, inhibitory control, gut hormones, brain structure, weight regain, and tissue-level and whole-body metabolism will be assessed.

ADAPT is a single group, longitudinal basic experimental study in humans [Fig 2]. It uses a behavioral weight loss program in participants with obesity to evoke entry into different phases of weight loss at which mechanistic outcomes are assessed [Fig 3]. The program is based on the PreventT2 curriculum [32,33] and involves group instruction and personalized coaching on reducing caloric intake to an individualized goal and increasing physical activity. Outcomes are assessed at study visits that are aligned with participants’ active weight loss and plateau phases. Study visit procedures provide comprehensive phenotyping of energy balance, behavior, and neurophysiological outcomes and include anthropometrics, behavioral questionnaires, physical activity monitoring via wearable device, daily remote weighing, functional and structural magnetic resonance imaging of the brain, fasting and serial blood draws during intake of a standardized meal, adipose tissue and muscle biopsies, indirect calorimetry, and body composition by dual x-ray absorptiometry (DXA). Planned analyses will compare bioenergetic, neurophysiologic, and hormonal outcomes between baseline and phases of weight loss. ADAPT takes place in the greater Puget Sound region of Washington state. In-person study visits occur at the University of Washington and Fred Hutchinson Cancer Center. All procedures used in this study adhered to the principles of the Declaration of Helsinki. The University of Washington’s Institutional Review Board approved of the study and its procedures with concurrence from the Fred Hutch Cancer Center IRB. All participants signed written informed consent.

### Remote weighing and real-time classification of phase of weight loss

A cornerstone of the ADAPT study design is the use of daily weighing to define the weight-loss phases in real time and schedule study assessments that align with participants’ individual periods of active weight loss and/or a weight loss plateau. Throughout the enrollment period, all participants are asked to weigh themselves daily on a study-provided electronic scale (BodyTrace™). Daily weights are remotely transmitted and monitored by the study. Participants are instructed to weigh themselves daily upon waking (fasted) and after voiding. Baseline weight is calculated as the average daily weight over the ~ 7-day period after the baseline visit, but before the start of the behavioral weight loss program. The baseline weight is used to calculate the daily percentage body weight change from baseline in real time.

Our pre-defined study criterion for active weight loss is based on the typically recommended pace of weight loss in a behavioral weight loss program and is defined as a rate of ≥0.5 lb/week. A weight loss plateau is defined as a rate within ±0.25 lbs/week. For each participant, daily percent weight change relative to baseline weight is calculated, and participant-specific slopes are derived that convert these percent changes into pound-per-week-equivalent rates aligned with the pre-defined study criteria for active weight loss and weight loss plateau.

**Fig 4:**
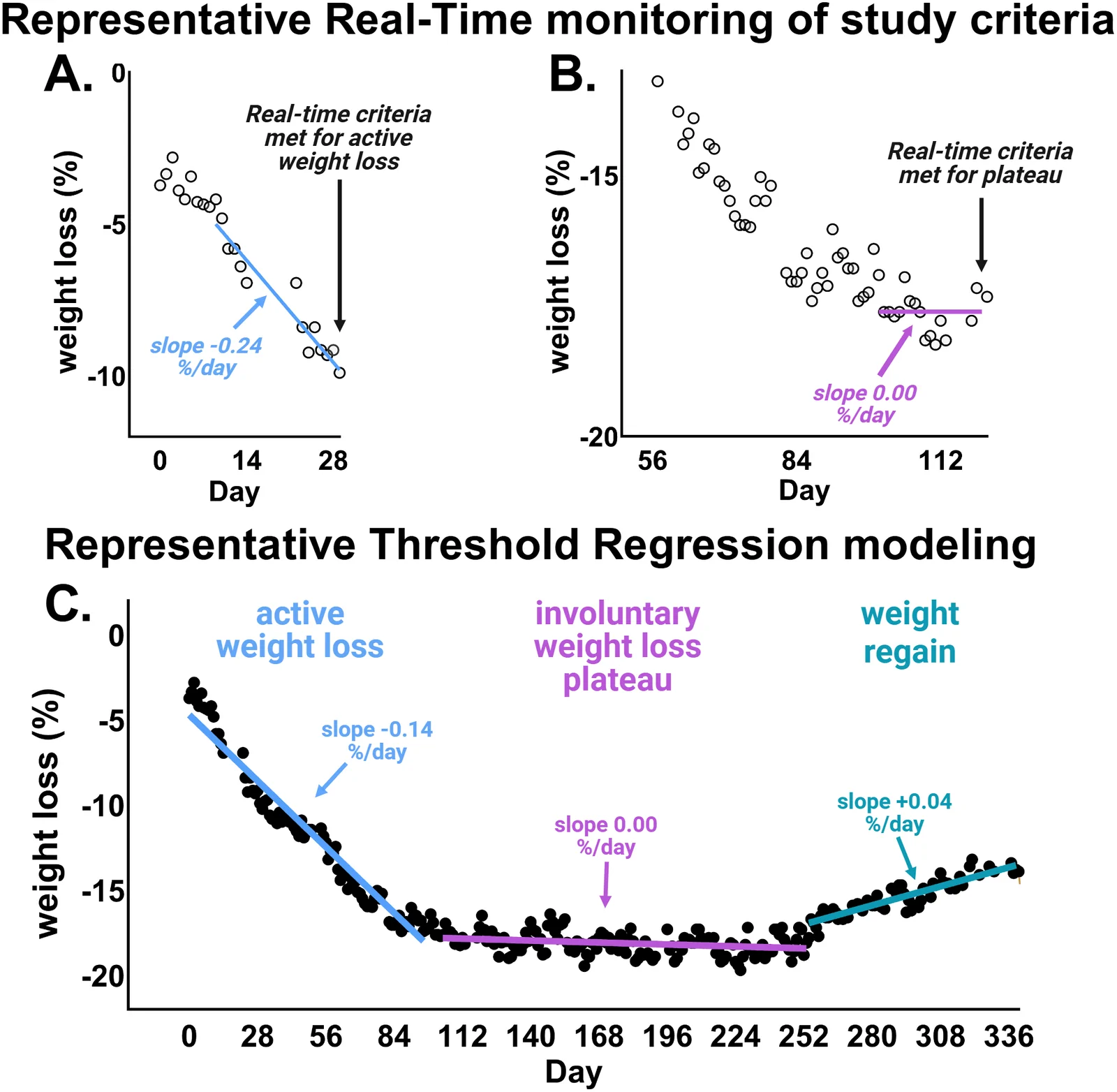
Representative example of real-time monitoring of study criteria for active weight loss and weight loss plateau and threshold regression modeling of an individual weight trajectory. In all panels, daily weights (empty and filled dots) are expressed as percentage change from baseline. A) Panel A depicts the example participant meeting active weight loss criteria in real-time when 1) their real-time 21-day slope (blue regression line; −0.24%/day) met or exceeded their personalized active weight loss slope criteria of −0.03%/day, and 2) a minimum of 4 weeks had elapsed since the start of the behavioral weight loss program. B) Panel B depicts the example participant meeting criteria for weight loss plateau visit when 1) they had previously met active weight loss criteria and 2) their real-time 21-day slope (purple regression line; 0.00%/day) met their personalized weight loss plateau slope criteria of within ±0.01%/day. C) Panel C depicts the example participant’s weight curve throughout the study with superimposed regression lines determined by threshold regression modeling. Threshold regression modeling uses all available weights in an unbiased characterization of phases of weight loss based on segmented slopes within the overall trajectory. The method is applied in two circumstances during study conduct: 1-2 months after completion of study visits (interim confirmation) and at study completion (final study phase assignment).

The real-time slope of daily percentage of weight change over the preceding 21-day period is monitored on a rolling basis. Participants who meet or exceed their personalized calculated active weight loss slope (equivalent to ≥0.5 lb/week) for the proceeding 21-day period are eligible to be invited for an active weight loss visit once they pass a minimum time threshold of 4 weeks of participation in the behavioral weight loss program. The 4-week minimum criterion is meant to avert acquiring study outcome measures during the early weight loss phase [19]. Participants are determined to be in an involuntary weight loss plateau when their real-time slope over the preceding 21-day period meets their personalized calculated plateau slope (equivalent to within ±0.25 lbs/week; See Fig 4 A and B for representative examples). Participants are only eligible to meet plateau criteria if they previously met active weight loss criteria. When participants meet either criterion, an in-person study visit for the appropriate visit type is scheduled within two weeks.

Threshold regression modeling of individual percent weight loss trajectories is performed as an adjunct analytic strategy to the real-time approach and is used for interim and final classification of participants’ individual weight-loss phase at the time study visits were conducted. Threshold regression modeling is a statistical method that integrates the additional repeated measures of weights accumulated after an in-person study visit and takes full advantage of both pre- (real-time) and post-visit data to establish the trajectory and slope of weight loss at the time the visit occurred. It allows retrospective assignment of weight loss phase at the time of an in-person visit using all available data. For example, a participant who met weight loss plateau criteria by demonstrating minimal weight loss over a 21-day period might subsequently resume losing weight more rapidly. In this situation, interim threshold regression modeling would take the post-visit weight loss into account to determine that the participant was not, in fact, in a physiologic plateau at the time of the assessment. If the interim threshold regression modeling fails to confirm that the in-person plateau visit occurred during a study-defined plateau phase, monitoring for plateau study-criteria would resume, and if criteria are met on a second occasion, the participant would be invited for a supplemental in-person plateau visit. Upon completion of the entire 18-month study period, threshold regression modeling is also applied to individual percent weight loss trajectories for final assignment of study phase for all in-person study visits. Statistical analyses will use the final study phase assignments. See Fig 4C for representative example.

### Eligibility

Eligible participants are age 18–60 years, BMI 30–50 kg/m^2^, able to attend the study’s behavioral weight loss program classes and study clinic visits for phenotyping assessments, and living independently with the ability to prepare their own meals. Exclusion criteria include: current use of cigarettes or any nicotine products; use of cannabis; heavy alcohol use (≥ 2 drinks/d for women and ≥ 3 drinks/day for men); recreational drug use; known cognitive impairment; prevalent or prior history of stroke, Type 2 diabetes, bariatric surgery, eating disorder, or other medical conditions that could limit ability to participate; current pregnancy/6 months postpartum or breastfeeding (women only); current use of medications with appetite suppressive effects or current anti-coagulants; current participation in formal weight loss program; current or prior participation in research study involving weight loss; weight reduction ≥10% in the past year; food allergy, intolerance or unwillingness to consume study foods provided at functional magnetic resonance imaging (fMRI) assessment; MRI contraindications; and lack of technology needed to complete study activities. Following informed consent, participants undergo a non-fasting blood draw (hemoglobin A1c, metabolic panel, complete blood count, thyroid stimulating hormone) and height, weight and vital measurements, then complete questionnaires for final eligibility determination.

### Recruitment

Participants from the greater Seattle, WA area are recruited through electronic, paper and social media advertisements into cohorts of 8–10 individuals. Interested participants complete an online screening form to determine initial eligibility followed by a telephone screen and an in-person visit to determine final eligibility. Recruitment began in December 2023 and is expected to end December 2026.

### Study visits

**Fig 5:**
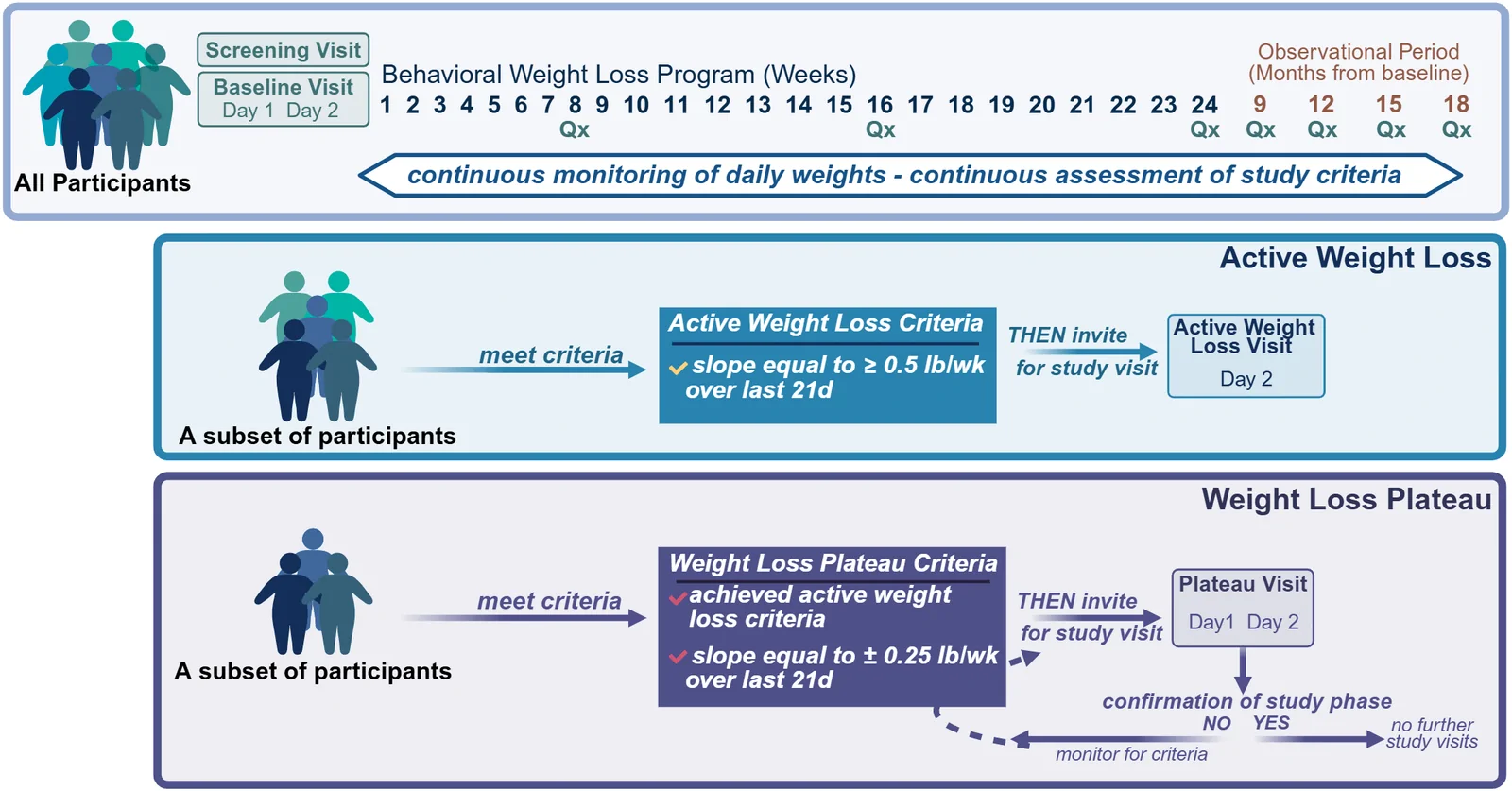
ADAPT study design for individualized outcome assessments and study visits corresponding to phases of weight loss. Following phone screening procedures, all participants complete in-person screening and baseline visits. After final eligibility is confirmed, all participants enter into the 24-week behavioral weight loss program, followed by a 1-year observational follow-up period. Remote questionnaires (Qx) are completed at week 8, 16 and 24 of the behavioral weight loss program, then during the observational period at 9-, 12-, 15- and 18-months from baseline. Daily weights are transmitted remotely to study staff after the baseline visit and continuously monitored throughout the 18-month enrollment period. Individual slopes of weight change are continuously calculated in real-time on a rolling basis over the previous 21-day period. Once a participant is determined to meet real-time criteria for an in-person study assessment, visits for the appropriate study phase are scheduled within 2 weeks of when the participant was identified as meeting study criteria. The participant may be invited to complete a supplemental plateau visit (occurring over 2 days) if interim threshold regression modeling fails to confirm a plateau phase at the time of their initial plateau visit.

**Fig 6:**
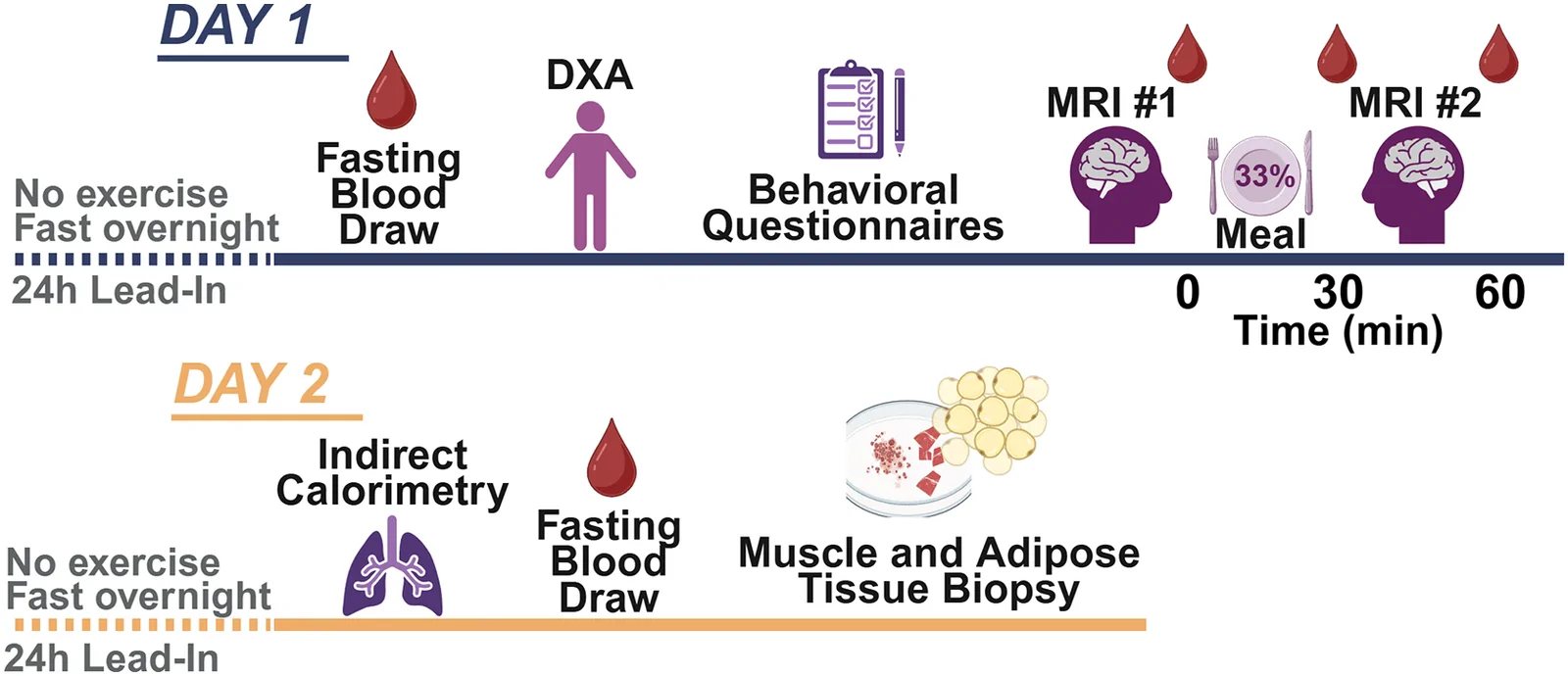
ADAPT In-Person Visit Day paradigms. Baseline and plateau visits occur over two days, active weight loss visits occur over a single day following the ‘Day 2’ paradigm. For all visit days, participants are asked to fast overnight and refrain from exercise for the previous 24 hours. The Day 1 paradigm begins with an IV placement, fasting blood draw, then anthropometric measures, vital sign measurements, and a dual x-ray absorptiometry (DXA) scan. Behavioral questionnaires, including cognitive assessments (i.e., General Cognitive Index and Go/No-Go) are completed. A fasted MRI (#1), including fMRI visual food cue task and structural MRI of the hypothalamus, is completed, then participants are presented with a standardized meal of macaroni and cheese and asked to consume it within 10 minutes. A fed MRI (#2) is completed 30 minutes after meal initiation and includes fMRI of visual food cues and resting state. Serial blood draws occur at time 0 (just prior to the standardized meal), then 30- and 60-minutes after the meal. Serial subjective appetite ratings by visual analog scale are administered throughout day 1 (i.e., upon arrival, after the DXA, prior to MRI #1, before and after the standardized meal and after MRI #2). Day 1 is completed at the University of Washington. The Day 2 paradigm begins with indirect calorimetry procedures, followed by a fasting blood draw and muscle and adipose tissue biopsies. Day 2 is completed at the Fred Hutchinson Cancer Center.

All participants undergo a baseline study visit and the 24-week behavioral weight loss program. They complete remote questionnaires at weeks 8, 16 and 24 of the program, then, during follow up at 9-, 12-, 15- and 18-months after the start of the behavioral weight loss program [Fig 5]. Baseline visits occur within approximately 30 days after the eligibility visit and procedures are completed on two separate study days. Participants are eligible to be invited to complete additional in-person visits if they meet the study-defined weight change criteria described above. In-person visit types triggered in real time based on participants’ individual weight loss trajectories include: active weight loss visits, weight loss plateau visits (occurs over 2 days), and supplemental weight loss plateau visits if the initial plateau is not confirmed by interim threshold regression modeling (as described above in *Remote weighing and real-time classification of phase of weight loss*). By nature of the study criteria, a plateau visit would occur at least 21 days after an active weight loss visit. Plateau and supplemental plateau visits follow the same two-day protocol as baseline visits, whereas active weight loss visits involve an abbreviated single-day protocol [Fig 6]. For all visit days, participants arrive following an overnight fast and having refrained from exercise within the past 24 hours.

As part of the Day 1 visit protocol, participants complete questionnaires and undergo weight, waist and neck circumference, vital sign measurements, and DXA. Serial blood draws occur throughout the visit day (AM fasting, pre-meal (also in a fasted state), 30- and 60-min post standardized meal). Cognitive assessments are completed on a desktop computer. Next, participants complete a pre-meal MRI session, consume a standardized meal, then a post-meal MRI session. Day 1 procedures are completed at the University of Washington. For the Day 2 visit protocol, completed at the Fred Hutch Cancer Center, indirect calorimetry procedures are completed, followed by a fasting blood draw and muscle and tissue biopsies. All procedure details are below.

### Behavioral weight loss program

The ADAPT weight loss program is based on the PreventT2 curriculum [33], an evidence-based lifestyle intervention that combines structured curriculum on nutrition and physical activity with goal-based behavior instruction to achieve weight loss, with modifications to condense the delivery of the program and incorporate additional instruction on the Harvard Healthy Eating Plate [34], healthy sources of dietary protein, and resistance exercise. The overall ADAPT study goal is a 10% reduction in baseline weight, to be met via a reduction in caloric intake and an increase in physical activity. Participants are provided an individualized calorie intake goal, which is calculated using the NIH Body Weight Planner [35], assuming 10% weight loss over 6 months with no change in physical activity, and using the participant’s age, height, weight and self-reported level of physical activity at baseline. Physical activity goals for all participants are 150 minutes per week of moderate intensity exercise and resistance training 2 times per week. Participants are asked to track their daily caloric intake using the MyFitnessPal app (free version) and their daily physical activity using the study-provided wearable device (see Activity Monitoring and Measurement section below).

The ADAPT weight loss program is delivered by a registered dietitian (RD) to a participant ‘cohort’ in group sessions. Over the 6-month program, participants complete a total of 16 sessions. After an initial one-on-one with the study RD to review individualized and ADAPT program goals, participants in the cohort meet as a group via HIPAA compliant videoconference for a total of 14 sessions. Group sessions occur weekly during weeks 2 through 8 (7 group sessions) and build the educational foundation of the program. In week 10, participants complete a second one-one-one with the study RD to review the participants’ progress to date and provide individualized information to help the participant overcome any barriers and achieve the study goals. In weeks 12–24, group sessions occur bi-weekly and emphasize supportive strategies to develop and maintain long-term healthy eating and physical activity habits. Each group session includes a check-in, review of concepts from the study session materials, and a facilitated group discussion. Follow up after each group session is provided by an email summarizing the study session concepts and group discussion topic/topics. During the weeks (8 total) when no group session occurs, a “toolbox” approach is used to provide supplemental information via email.

### Data collection procedures

#### Clinical measurements.

At all visit types, anthropometrics and vital signs are measured by standard methods. Height is measured on a wall-mounted stadiometer (screening only). The average of two measures is calculated (if > 0.5 cm discrepancy exists, a 3^rd^ measure is obtained). Heart rate, blood pressure and temperature are measured around the same time of day (morning) and after at least a 5-minute rest period by standard electronic methods. Weight measures are done in duplicate on a standing electronic scale and are to be within 0.1 kg (or a third measure is taken). Waist circumference is measured in duplicate after the participant removes their shoes. Briefly, the abdomen is exposed, and the participant stands with their feet hip-width apart and weight evenly distributed; after crossing their arms in front of them, study-staff locate their right upper hip bone and upper iliac crest and note the midaxillary line. A spring-loaded tape measure is placed around the participant’s abdomen at the level of the determined iliac crest. Two measurements are taken during exhalation. If they are > 0.5 cm different, a third measure is completed. Neck circumference is completed in duplicate within 0.5 cm, at the trunk of the neck. Averages of duplicate measures are calculated. Staff cross-train on measurement procedures to ensure similar approaches between staff and study visit sites. Body composition is measured by DXA (iDXA; GE Healthcare, Madison, WI) and abdominal visceral adipose tissue mass (VAT) determined with Encore software (version 14.10). A fasting blood draw is completed at all visits. Additional serial samples are obtained by indwelling IV catheter during Day 1 visits just prior to a standardized meal, then 30- and 60-min after the meal. Plasma and serum are aliquoted after standard processing and stored at −80°C for planned measures.

#### Magnetic resonance imaging and the standardized meal.

Participants undergo two MRI sessions as part of the Day 1 visit protocol. The pre-meal/fasting session includes quantitative structural imaging and fMRI while viewing visual food cues. The Fred Hutchinson Human Nutrition Laboratory prepares a standardized meal of macaroni and cheese (~40% carbohydrate, ~ 40% fat, ~ 15% protein) for the participant to provide ~33% of their estimated daily caloric need calculated by Mifflin and a standard activity factor of 1.2. Participants are asked to consume the meal within 10 minutes. The second MRI session includes fMRI of visual food cues and resting state, and occurs approximately 30 minutes after meal initiation.

*MRI acquisitions.* All scans are acquired with a 32-channel SENSE head coil on a 3-Telsa Philips Ingenia CX with dual Quasar gradients. In both sessions, a 306 volume T2*-weighted single-shot echo-planar imaging (EPI) time series is acquired during passive picture viewing. At the post-meal session, a 372 volume T2*-weighted single-shot EPI time series is acquired during rest (viewing of a crosshair). Both EPI acquisitions include: 51 ascending axial slices, 2.50 mm isotropic voxels, repetition time (TR)= 1200 ms, echo time (TE) = 30ms, flip angle = 70°, multi-band SENSE factor = 3. For distortion correction and registration a B0 field map (TR = 6.2ms; TE = 3.1ms; delta TE 1.0 ms; flip angle = 10°) and a compressed-sense 3D Magnetization-Prepared Rapid Gradient-Echo image with 176 sagittal slices (TR = 7.6, TE = 3.5 ms, flip angle = 7°, CS-SENSE factor = 3, matrix 240 x 240, 1 mm isotropic voxel) are acquired. A quantitative multi-slice/multi-echo T2-weighted sequence with 16 echoes (interecho spacing 10ms, slice thickness 2.0 mm-no gap) is acquired fasting. Coronal slices are acquired through the hypothalamus from the optic chiasm to the mammillary bodies.

*fMRI paradigm.* Details of our established fMRI protocol, procedures, stimuli, acquisition parameters, and processing are published elsewhere [36–38], final fMRI preprocessing protocols will be made publicly available online. Briefly, previously-validated stock photographs included high-calorie foods (e.g., pizza, desserts), low-calorie foods (e.g., fruits, vegetables) and non-food objects (e.g., household items, sports equipment). All food images are ready-to-eat, and non-food objects are easily recognizable. A subset of images are scrambled to be unrecognizable but retain similar color qualities. Each fMRI session includes a distinct set of 15 blocks with 10 images each presented for 2.4 seconds. Non-food blocks (N = 6) and scrambled image blocks (N = 3) are interleaved with high-calorie (N = 3) and low-calorie (N = 3) food blocks. The order is counterbalanced across participants (half viewed low-calorie images as the 1^st^ food block). All images are displayed on a dark gray background, were equal in size (600x400 dpi) and were matched for luminosity (F=(3,56)=0.26, P = 0.85) and food sub-category (e.g., comparable number of fruit and vegetable images) across blocks.

Participants are instructed to remain awake throughout the MRI sessions. Short videos are played during structural acquisitions. Prior to the fMRI tasks, participants are instructed to “think about how the food would taste if you ate it or how the object would feel to touch”. For the fMRI resting state, a white cross-hair is displayed on a black background and participants are asked to “focus on the cross, relax and let your mind wonder”. To assess attention during the visual food cue task, after each session participants are asked to respond yes or no if they recalled seeing a sub-set of 32 images in the scanner. Half of the images presented are distractor images and not displayed during the task.

*fMRI analytic approach.* As noted above, participants undergo fMRI before and after a standardized meal during which they view images of high-calorie food, low-calorie food, non-food objects and scrambled images. As a measure of central satiety response, planned initial analyses will measure activation within a set of *a priori* regions of interest (ROIs) established as markers of satiety [38,39] or predictors of food choice [38] (amygdala, insula, dorsal striatum, ventral striatum, medial orbitofrontal cortex, ventral tegmental area/substantia nigra) and shown by our group to be altered after behavioral obesity treatment in children [10]. A second set of inhibitory control ROIs will include the dorsolateral prefrontal cortex, inferior frontal gyrus, and posterior parietal lobe. These regions play important roles in exertion of dietary control [40] and value modulation [41], as well as response inhibition [42], and participate in functional executive-control networks [43,44] that are engaged to inhibit actions in accordance with immediate or long-term goals [40,42]. An additional ROI in the ventromedial prefrontal cortex will be included as it appears to be the downstream target of inhibitory control regions and may mediate computation of reward value during self-regulation [45,46]. ROIs will be created using anatomic-functional criteria [47]. Individual mean parameter estimates will be extracted from each ROI and then averaged. The primary contrast will be high-calorie food vs. object images. Comparison contrasts will be low-calorie food vs. objects and high-calorie vs. low-calorie food images. The use of ROIs provides an unbiased approach as well as transparency (they will be pre-determined and published) and reproducibility, critical elements to ensure rigor in fMRI studies [48]. Voxel-wise analyses of regions outside *a priori* ROIs will be considered exploratory.

*Structural MRI of the hypothalamus analytic approach*. We have validated T2 relaxation time as a marker of mediobasal hypothalamic gliosis in rodents [49] and humans [50]. Multi-slice/multi-echo T2-weighted sequences [50,51] are obtained at baseline and plateau. The coronal slice immediately posterior to the optic chiasm will be identified, regions of interest including the mediobasal hypothalamus and two reference regions (amygdala and putamen) will be placed bilaterally on high-resolution images, then transferred to the T2 parametric map and mean T2 relaxation time will be extracted for each region of interest.

#### Questionnaires and Behavioral Assessments.

Participants complete questionnaires prior to and at each in-person visit, and remotely at weeks 8, 16 and 24 of the behavioral weight loss program, then 9-, 12-, 15- and 18-months following the start of the program. Questionnaires assess medical history, sleep, physical activity, and eating behaviors. See Table 1 for a complete list of questionnaires, descriptions, and timing of assessments.

**Table 1:** Questionnaires.

Questionnaire	Description, measurement	Baseline	Serial assessment^#^	Active Weight loss	Plateau
			Week 8, 16, 24 Month 9, 12, 15, 18	If applicable	If applicable
Medical history	Self-report on major medical history and current medications	*Completed at screening*	*Reviewed* *at 9M and 18M*		*Reviewed*
Demographics	Self-report of sex, age, race and ethnicity, employment status, education level	X			
State-Trait Anxiety Inventory-Trait (STAI-Trait) [52]	Trait (general) components of anxiety	X			
Patient Health Questionniare-9 (PHQ-9) [53]	Depressive symptoms	X			X
STOP-Bang [54]	Screening for obstructive sleep apnea	X			X
Pittsburgh Sleep Quality Index (PSQI) [55]	Sleep quality	X			X
International Physical Activity Questionnaire (IPAQ)	Amount and level of physical activity during previous 7 days (short form)	X		X	X
Women’s Health Questionnaire	Reproductive and menstruation history in all female participants, *study-generated*	X		X	X
**Eating Behavior**					
Food Frequency Questionnaire (FFQ)*	Dietary Intake over last month	X		X	X
Subjective Appetite^$^	Visual analog scale, assessed throughout in-person visit	X		X	X
Mindful Eating Questionnaire (MEQ), 28-item [56]	Awareness of physical and emotional sensations associated with eating	X			X
Three-Factor Eating Questionnaire (TFEQ), 18-item [57]	Subscales: Cognitive restraint, uncontrolled eating, emotional eating	X	X	X	X
Reward-Based Eating Drive Scale-13 (RED-13), 13-item [58]	Reward-related eating behavior	X	X	X	X
Food Craving Inventory-II (FCI-II), 33-item [59]	Frequency of cravings with 5 subscales	X	X	X	X
Weight Efficacy Lifestyle-short form (WEL-SF), 8-item [60]	Self-confidence for controlling eating behavior during weight management	X	X	X	X

*Dietary intake data are collected using a food frequency questionnaire developed by the Nutrition Assessment Shared Resource (NASR) of Fred Hutchinson Cancer Center, Seattle, Washington. Nutrient calculations are performed using the NDSR software version 2024 (Nutrition Coordinating Center, University of Minnesota, Minneapolis, MN). ^**#**^Serial assessments are completed remotely throughout the enrollment period at week 8, 16 and 24 of the behavioral weight loss program, then at 9-, 12-, 15-, and 18-months from the start of the program. ^$^Subjective appetite is measured with serial visual analog scales to assess hunger and fullness during Day 1 visits. Questions, such as ‘How hungry to you feel’, are presented on an iPad with a sliding scale and include anchors such as ‘I am not hungry at all’ and ‘I have never been more hungry’. The electronic sliding scale measures responses from 0-100 mm. Other VAS questions measure desire to eat, prospective food consumption, tiredness and thirst. VAS assessments are completed upon study visit arrival, after the DEXA, before MRI session #1, before and after the standardized meal, and after MRI session #2.

*Behavioral Tasks*. Participants complete the General Cognitive Index and Go/No-Go behavioral tasks in-person at baseline and remotely at 18-months. If applicable, both are also completed in-person at active weight loss, plateau and supplemental plateau visits. The General Cognitive Index (testmybrain.org, NIH-General Cognitive Index – Matrix Reasoning and Digit Symbol Matching) is completed on an online platform and measures general cognition. A Go/No-Go (testmybrain.org; TMB Gradual Onset Continuous Performance Test) task is completed to measure response inhibition.

#### Activity monitoring and measurement.

Participants are provided with a FitBit Inspire 3 device (Fitbit Inc., San Francisco, CA) that is linked to the study’s Fitabase (Fitabase, San Diego, CA) platform for monitoring and data collection. Participants are asked to wear the device continuously and sync it to their app daily throughout their study participation, beginning at the screening visit. The study captures data continuously from baseline through the end of the behavioral weight loss program, then in month-long units at each follow-up timepoint (9-, 12-, 15-, 18-months). Outcome measures include frequency of activity (min/d), activity intensity (moderate, vigorous, peak), total steps/day, average resting heart rate, average heart rate during activity, and heart rate variability.

#### Indirect calorimetry.

Indirect calorimetry using a metabolic cart (MGC diagnostics) is performed on Day 2 of the baseline and plateau visits, and at the active weight loss visit. Participants are instructed to remain still with minimal movement and without sleeping and a fitted mask or canopy is placed securely over the nose and mouth to ensure that no gas escapes during the test. The participant rests for up to 30 minutes in a supine position to acclimate to the apparatus, then an approximate 20-minute test is performed to achieve steady state. Data from the indirect calorimetry are used to calculate respiratory quotient (RQ) and resting energy expenditure (REE).

#### Biopsies.

Adipose and muscle biopsies are conducted by qualified personnel on day two of the Baseline and Plateau visits and at the active weight loss visit. Following injection of local anesthetic, adipose biopsies are collected using negative pressure from an incision site located in the lower quadrant of the abdominal area, 10–12 cm from the umbilicus. To provide superficial anesthetic, up to 10 mL of 1% lidocaine is injected into the dermis of the target area. Once the anesthetic has taken effect and the site is sufficiently numb, a < 0.5 cm scalpel incision is made through the adipose tissue, and pressure is applied with gauze to stop bleeding. A biopsy needle is passed through the incision into the subcutaneous fat, and negative pressure is applied to obtain the sample. Pressure is applied to the incision with gauze while the sample is removed from the biopsy needle and placed on ice. The wound is then closed with steri-strips while the adipose biopsy sample is weighed and portioned into two cryovials, both of which are immediately flash frozen using liquid nitrogen then stored at −80°C until analysis.

Also under local anesthetic, muscle biopsies are collected via an incision over the vastus lateralis muscle, 8–10 cm proximal to the knee, lateral to midline, and medial to the IT band. To provide superficial anesthetic, up to 5 mL of 1% lidocaine is injected into the dermis of the target area. After the initial anesthetic injection, 1% lidocaine is again injected and advanced into the quadricep muscle, distributing the anesthetic into the muscle belly. Once the anesthetic has taken effect and the site is sufficiently numb, a number 11 blade scalpel is used to make an incision through the skin, subcutaneous tissue, fascia, and into the vastus lateralis muscle. A biopsy needle is inserted into the incision, through the fascial layer and is advanced an additional 2–3 cm past to ensure the biopsy needle is positioned completely within the muscle belly. Negative pressure is applied to obtain the sample, then pressure is applied to the incision with gauze while the sample is removed from the biopsy needle and placed on ice. The wound is then closed with steri-strips while the muscle biopsy sample is weighed and portioned into four cryovials; one cryovial is immediately flash frozen using liquid nitrogen and the remaining samples are transported on ice for processing and analysis.

### Measurement of Biospecimens

#### T-cell isolation and high-resolution respirometry.

Because peripheral blood mononuclear cells (PBMCs) represent a heterogeneous population of cells, T-cells are selected from freshly isolated PBMCs as previously described [61]. Three million cells are resuspended in supplemented DMEM (5 mM HEPES, 5 mM glucose, 1 mM sodium pyruvate, and 2 mM glutamate), and four million cells are resuspended in Mir05 (0.5 mM EGTA, 3 mM MgCl_2_, 60 mM lactobionic acid, 20 mM taurine, 10 KH_2_PO_4_, 20 mM HEPES, 110 mM D-sucrose). High resolution respirometry is performed using an Oroboros O2K respirometer (Oroboros Instruments, Austria) in intact cells (Protocol 1) and permeabilized cells (Protocol 2). For Protocol 1, T-cells are injected into chambers containing supplemented DMEM. Once basal respiration becames stable, leak respiration is induced with 1mM oligomycin, maximal uncoupled respiration is stimulated with consecutive titrations of 0.5 mM 2-(trifluoromethoxy) phenyl hydrazinylidene-propanedinitrile (FCCP), and complex III is inhibited with 2.5 μM antimycin A to induce non-mitochondrial respiration. For Protocol 2, T-cells are injected into chambers containing Mir05 supplemented with 1 μM of TMRM to measure membrane potential as previously described [61]. Cells are permeabilized with 4 μg/mL of digitonin. Leak respiration and maximal mitochondrial membrane potential is stimulated with 5 mM succinate, then 10 mM glutamate, 1 mM malate, and 5 mM pyruvate. Adenosine di-phosphate (ADP, 1 mM – 4 mM) is then titrated to induce oxidative phosphorylation. Maximal uncoupled respiration is then stimulated by sequential 0.5 mM FCCP injections. Complex I is inhibited with 0.5 mM rotenone, and Complex III with 2.5 mM antimycin A. Non-mitochondrial respiration under antimycin A is subtracted from all respiration rates for statistical analysis.

### Permeabilized muscle fiber preparation and high-resolution respirometry

A small piece (~15 mg) of fresh muscle tissue is immediately placed into ice-cold BIOPS (10 mM Ca-EGTA, 0.1 mM free calcium, 20 mM imidazole, 20 mM taurine, 50 mM K-MES, 0.5 mM dithiothreitol, 6.56 mM magnesium chloride, 5.77 mM sodium adenosine triphosphate, 15 mM phosphocreatine, pH 7.1). Tissue is manually teased with microdissection forceps into small fiber bundles of ~ 2 mg and permeabilized in ice-cold BIOPS with saponin (30 ug/mL) for 30 minutes and rinsed with BIOPS and Mir05 for 25 minutes. One fiber bundle is placed in a O2K chamber containing Mir05 supplemented with 25 mM blebbistatin. Protocol 3 includes a series of injections to induce respiration driven by oxidative phosphorylation of fatty acids, complex I and II substrates (Protocol 3: 4 mM ADP, 0.1 mM malate and 0.2 mM octanoyl-carnitine, 10 mM cytochrome C, 15 mM palmitoyl carnitine, 5 mM glutamate, 0.5 mM malate, 20 mM pyruvate, 10 mM succinate). Another fiber bundle is placed in a 2-mL O2K chamber containing Mir05 + blebbistatin and 10mM Amplex Ultra-Red reagent containing 5 U/mL superoxide dismutase and 1 U/mL horseradish peroxidase. Protocol 4 includes the addition of 10 mM succinate, 5 mM glutamate, 0.5 mM malate and 5 mM pyruvate to induce reverse electron transport and leak state. Adenosine di-phosphate (ADP) was then titrated to induce oxidative phosphorylation through a range of ADP concentrations (1 mM – 4 mM). Maximal uncoupled respiration is then stimulated by sequential 0.5 mM injections for protocols 3 and 4. Non-mitochondrial respiration under 2.5 μM antimycin A is subtracted from all respiration rates for statistical analysis. Oxygen is maintained at 500−200 mM uM.

#### Mitochondrial respiration in white adipose tissue (WAT) samples.

Approximately 100 mg of frozen WAT is used for the mitochondrial respiration assays. Samples are thawed on ice in mitochondrial assay solution (MAS), and the tissue is minced into smaller pieces with scissors. The fragments are collected and subsequently homogenized in ice-cold MAS. Mitochondria-enriched homogenates are obtained by differential centrifugation steps to remove the fat layer and other debris. Protein concentration is determined using the Pierce Rapid Gold BCA Protein Assay (Thermo Fisher Scientific).

Mitochondrial respiration in frozen samples is assessed using the Agilent Seahorse XFe24 Analyzer (Agilent Technologies, Santa Clara, CA) as previously described with minor modifications [62,63]. Homogenates containing mitochondria, and supplemented with cytochrome c, are loaded into Agilent Seahorse XF24 Cell Culture Microplates. For assessments of Complex I, II, and IV activities, substrates and inhibitors are injected subsequently as follows: NADH or Succinate + Rotenone (port A), rotenone + antimycin A (port B), TMPD + ascorbic acid (port C), and sodium azide (port D). Oxygen consumption rates are normalized to mitochondrial content.

### Retention/compliance/adherence

Adherence to the weight loss protocol is measured by attendance to behavioral weight loss program sessions (number of group and one-on-one sessions attended), dietary tracking (number of days per week the participant tracked >=500 kcal) and physical activity tracking (number of days the participants’ FitBit device was synced).

### Data management and availability

All participants are assigned a unique study ID upon enrollment, and all data is de-identified at the point of data collection. Data is directly electronically captured in RedCap when possible (e.g., questionnaires), otherwise data is entered into RedCap (e.g., clinical measurements) by study staff, or maintained on secure servers in its native form until processing (e.g., fMRI data) or integration into the study database. Data not directly captured is doubled data entered and checked for discrepancies. Data will be available from the study, once completed, upon reasonable request to the principal investigators. Required details and results will be reported to clinicaltrials.gov and further dissemination of results will occur through scientific conferences and publications.

### Statistical analysis plan

#### Aim 1.

Primary outcomes are oxidative phosphorylation and maximal respiratory capacity in T-cells, and permeabilized muscle fibers, the latter with fatty acid oxidation and complex I substrate. Secondary outcomes include T-cell and permeabilized muscle fibers uncoupling (proton leak respiration), adipose tissue mitochondrial respiratory capacity and coupling, UCP-1 expression, and inflammatory and lipolytic markers. As a prelude to inferential testing, linear regression will assess change in each measure as a function of potential confounding baseline covariates including % body fat, age, and sex as well as resting energy expenditure. Linear mixed models (adjusted for baseline confounders if needed) will be used to test for a main effect of time (baseline, active weight loss, plateau), followed by Bonferroni-adjusted formal *post hoc* testing to assess differences between phases and evaluate if a weight loss plateau represents a distinct metabolic state. We will then explore *evidence of cross talk* among T-cells, muscle, adipose tissue, and brain measures of appetite (See Aim 2 below). For measures that are changed from baseline by weight loss, we will perform linear regression amongst outcomes of the change from baseline to active weight loss or weight loss plateau. *Implications for weight maintenance* will be explored via linear regression of % weight change from plateau to 18 mo. as a function of the change in primary and secondary metabolic measures at plateau phase from baseline, adjusting for time between plateau and end of follow-up. Secondary analyses will use values measured at plateau (not change). Stratified analyses will assess sex as a biological variable. A sample size of N = 40 at active weight loss and plateau provides 99% power to detect within-subject differences between baseline and plateau and end of follow up for primary outcomes.

#### Aim 2.

The primary analyses will evaluate differences between baseline and plateau phase in brain activation to visual food cues, specifically: 1) meal-induced change in mean extracted parameter estimates across all *a priori* satiety regions (high-calorie food vs. objects), 2) changes in pre-meal activation (high-calorie food vs. objects) from each of the four inhibitory control and reward modulation regions, and 3) associations of any findings with changes in Aim 1 metabolic outcomes. Outcomes for secondary analyses include mediobasal hypothalamic T2 relaxation time (vs. control regions) and meal-induced changes in salience network connectivity. Associations with weight regain post-plateau will be explored as will within-subject differences in regions outside *a priori* ROIs via fully adjusted voxel-wise comparisons. For within-subject comparisons, we will use linear mixed models that can accommodate repeated measures as well as missing data. Relationships between neurophysiologic measures and behavioral and hormonal correlates will be assessed using linear regression of change in behavioral correlates from baseline to plateau, as a function of each measure separately and together as covariates (i.e., changes in satiety response and inhibitory control activation), adjusting for time between plateau and baseline and, as needed, baseline covariates that are potential confounders such as sex, age, and % body fat. A sample size of N = 43 at plateau maintains 80% power to detect within subject differences in brain activation to visual food cues.

#### Aim 3.

The primary outcomes of interest being explored in Aim 3 are entry into a weight loss plateau (Yes/No) and time from baseline (beginning of behavioral weight loss program) to plateau entry (continuous). Entry into a weight loss plateau will be defined as the 1) day at which a subject met weight loss plateau criteria during real-time monitoring or 2) first day of a weight loss plateau identified via threshold regression modeling at study completion. *A priori* predictors of interest for Aim 3 are based on available literature and our own findings and include the slope of the first active weight loss period [64]; trends over time in craving for carbohydrates/starches, dietary restraint, and disinhibition [65]; changes in T-cell and muscle maximal respiratory capacity; fasting ghrelin [66]; and baseline central satiety response, mediobasal hypothalamic T2 relaxation time [67], leptin [28] concentrations, and HOMA-IR [68]. Other predictors (i.e., inhibitory control) may be tested based on the findings of Aims 1 and 2; remaining predictors will be considered exploratory. Analyses will include only participants who achieved active weight loss. Fully adjusted models will include baseline or change in % body fat. Sensitivity analyses will be completed to evaluate whether excluding participants not achieving adherence will have the same or better predictive capabilities. A sample size of N = 40 participants who achieve active weight loss provides 91% power to detect effect sizes of R^2^ ≥ 0.36 at P < 0.05 for continuous outcomes.

## Discussion

The ADAPT study is poised to make novel contributions to scientific understanding of volitional weight loss. It implements unique design elements that assess tissue-level bioenergetics as well as neurophysiologic and behavioral outcomes. These elements address gaps in the literature and could provide insights into mechanisms resulting in an involuntary weight loss plateau. Consequently, findings could be informative in several respects. They could suggest modifications to lifestyle change recommendations to extend weight loss, uncover potential molecular targets involved in modulating energy consumption and efficiency during weight loss, or identify neural pathways that underlie the appetitive responses that can resist weight loss or produce symptoms of hunger, loss of satiety or pervasive thoughts of food. Any of these discoveries could be further investigated to determine their role in the occurrence of weight loss plateaus during other modes of treatment, including metabolic surgery or pharmacologic treatments. Additionally, the findings could be applied to better understand physiological drivers of weight maintenance and to identify ways of supporting long-term weight maintenance. In sum, the ADAPT study shows potential to advance therapeutic options for prolonging the weight loss phase, attaining a greater degree of weight loss when it is required for health benefits, and contributing to knowledge of the aspects of weight loss that improve health outcomes for patients.

## Supporting information

S1 File SPIRIT checklist. (DOCX)
